# Supplementary material for: Normal mitochondrial function in Saccharomyces cerevisiae has become dependent on inefficient splicing
Source: eLife. 2018 Mar 23;7:e35330. doi: 10.7554/eLife.35330 (PMC5898908; doi:10.7554/eLife.35330)
Supplement: Transparent reporting form [file elife-35330-transrepform.docx]

***eLife’s* transparent reporting form**

We encourage authors to provide detailed information *within their submission* to facilitate the interpretation and replication of experiments. Authors can upload supporting documentation to indicate the use of appropriate reporting guidelines for health-related research (see [EQUATOR Network](http://www.equator-network.org/%20)), life science research (see the [BioSharing Information Resource](https://biosharing.org/)), or the [ARRIVE guidelines](http://www.plosbiology.org/article/info:doi/10.1371/journal.pbio.1000412) for reporting work involving animal research. Where applicable, authors should refer to any relevant reporting standards documents in this form.

If you have any questions, please consult our Journal Policies and/or contact us: [editorial@elifesciences.org](mailto:editorial@elifesciences.org).

**Sample-size estimation**

- You should state whether an appropriate sample size was computed when the study was being designed
- You should state the statistical method of sample size computation and any required assumptions
- If no explicit power analysis was used, you should describe how you decided what sample (replicate) size (number) to use

Please outline where this information can be found within the submission (e.g., sections or figure legends), or explain why this information doesn’t apply to your submission:

Information on the replicate number, as well as the number of cells used for the replicative lifespan measurements can be found in the Methods section, as well as in the Figure Captions.

**Replicates**

- You should report how often each experiment was performed
- You should include a definition of biological versus technical replication
- The data obtained should be provided and sufficient information should be provided to indicate the number of independent biological and/or technical replicates
- If you encountered any outliers, you should describe how these were handled
- Criteria for exclusion/inclusion of data should be clearly stated
- High-throughput sequence data should be uploaded before submission, with a private link for reviewers provided (these are available from both GEO and ArrayExpress)

Please outline where this information can be found within the submission (e.g., sections or figure legends), or explain why this information doesn’t apply to your submission:

The numbers of cells curated for the determination of replicative lifespan are listed in Figure S4 caption. Replicates for the determination of the phenotypic response to intron absence are defined in Figure 1 caption (for the wild type background), Figure 3 caption (for the Rtg2-null background), Figure 4 caption (for the Hap4-null background) and Figure 5 caption (for the evaluation of transcript level effects). This includes replicates for the measurements of oxygen consumption, mitochondrial mass and inner mitochondrial membrane potential, ATP levels, as well as ROS levels. Replicates for the measurement of transcriptional response (qPCR) to intron absence are defined in the Figure 2 caption. The number of cells that were analyzed for mitochondrial morphology is noted in the Methods section, as well as the number of cells curated for counting of foci related to *cox1* introns and exons in the RNA FISH experiment.

**Statistical reporting**

- Statistical analysis methods should be described and justified
- Raw data should be presented in figures whenever informative to do so (typically when N per group is less than 10)
- For each experiment, you should identify the statistical tests used, exact values of N, definitions of center, methods of multiple test correction, and dispersion and precision measures (e.g., mean, median, SD, SEM, confidence intervals; and, for the major substantive results, a measure of effect size (e.g., Pearson's r, Cohen's d)
- Report exact p-values wherever possible alongside the summary statistics and 95% confidence intervals. These should be reported for all key questions and not only when the p-value is less than 0.05.

Please outline where this information can be found within the submission (e.g., sections or figure legends), or explain why this information doesn’t apply to your submission:

Statistics regarding the analysis of the data in this study is described in the Methods section as well as in Figure Captions.

(For large datasets, or papers with a very large number of statistical tests, you may upload a single table file with tests, Ns, etc., with reference to sections in the manuscript.)

**Group allocation**

- Indicate how samples were allocated into experimental groups (in the case of clinical studies, please specify allocation to treatment method); if randomization was used, please also state if restricted randomization was applied
- Indicate if masking was used during group allocation, data collection and/or data analysis

Please outline where this information can be found within the submission (e.g., sections or figure legends), or explain why this information doesn’t apply to your submission:

Masking was not used during data analysis.

**Additional data files (“source data”)**

- We encourage you to upload relevant additional data files, such as numerical data that are represented as a graph in a figure, or as a summary table
- Where provided, these should be in the most useful format, and they can be uploaded as “Source data” files linked to a main figure or table
- Include model definition files including the full list of parameters used
- Include code used for data analysis (e.g., R, MatLab)
- Avoid stating that data files are “available upon request”

Please indicate the figures or tables for which source data files have been provided:

Supplemental Table 1 contains a full list of genes and corresponding primer pairs used for the qPCR measurement of the transcript levels, sequences of primers used for cloning, as well sequences of Stellaris probes used for the RNA FISH experiment.
